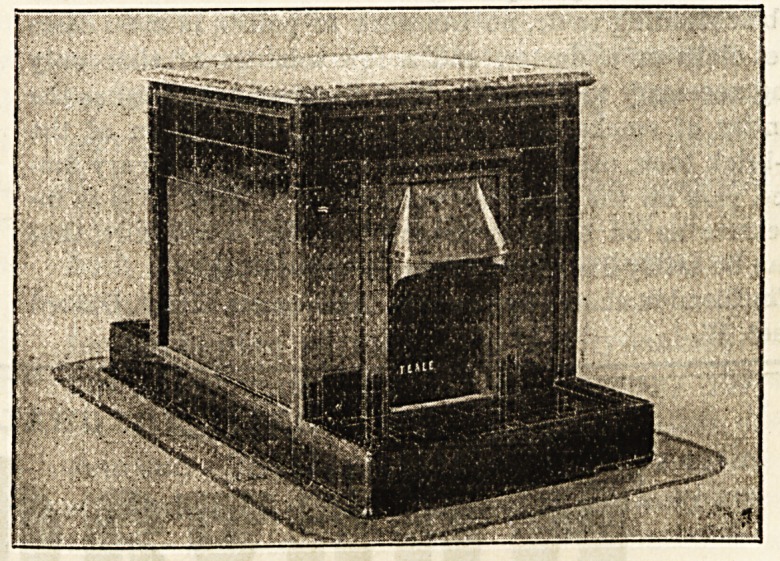# Practical Departments

**Published:** 1903-07-25

**Authors:** 


### PRACTICAL DEPARTMENTS.

### TEALE FIREPLACES.

The fireplaces manufactured by Messrs. Lionel H. Teale and R. Mousley are deservedly making a name for themselves in the annals of hospital construction, the principles on which they base their claim being mainly three—cleanliness, economy, and artistic appearance.

The stoves are the result of many years' experiment. It was in 1886 that Dr. T. Pridgin Teale, F.R.S., lectured before the members of the Royal Institution on the principles of fireplace construction, and his "economiser," fitted to old stoves for the prevention of waste heat, was subsequently developed into the original Teale fireplace with steel bars, known as the "Economiser." It is constructed almost entirely of fire-brick, with a hot-air chamber below the fire, which burns in a hollow or well.

The "Front Hob" fireplace is constructed entirely of fire-brick, with the exception of the grate bottom and the ash pan, which could hardly be made of anything but iron. The fuel, therefore, is surrounded on all four sides by firebrick, about three inches of this material being laid on the raised hearth, which is the special feature of the stove. The fire burns in a well, as in the "Economiser," but the bars are replaced by the hearth, or hob, which makes an excellent hot plate, besides radiating the heat out into the room. The ashes are collected in a pan which is readily withdrawn for emptying. These fireplaces are made in a variety of patterns, with wood or tiled overmantels, copper or steel ornaments; while in some cases metal enamel medallions are brought into use to heighten the artistic effect. Dutch tiles in blue and white are particularly pleasing, while Spanish lustre in gold and green, or gold and brown, gives a very comfortable glow throughout a room. The decorative side of the work is in the hands of an experienced artist.

So far we have spoken of fireplaces suitable for private institutions and nursing homes, such as have been supplied to the Home Hospital, Fitzroy Square, and many other well-known institutions, including the new Nurses' Home at the Middlesex Hospital.

Our illustration, however, shows the Teale stove in use at the London and many other hospitals for warming the wards. It is a back-to-back fireplace, designed to secure the maximum of heat without waste. The flues, which are separate for the two stoves, are carried either up through the ceiling or down through the floor, the latter having this advantage, that it allows a full and unbroken view of the ward, while the top of the stove admits of artistic tiling and can be used more or less as a table. There is a ventilator under these top tiles, which, not being apparent to a person standing upright, is no disfigurement. It is designed to ensure the warming of the air passing through it, and it is claimed that the temperature of a ward thus heated is sufficiently warm throughout every part, as well as near the stove. In warm weather one stove only need be used.

The following are some of the advantages claimed for the Teale fireplace:—

That it will burn throughout a night; indeed from 12 to 24 hours without replenishing the fuel, or any attention whatever. That it is an ideal fireplace for a sick-room, as the ashes can be removed without disturbing the fire. That it frequently cures a smoky chimney and never makes a bad chimney worse. That it will burn any kind of fuel—coal, coke, peat, wood, and, by a slight modification, anthracite. That it will effect an enormous saving in the expense for fuel, estimated at about one-third. That it diminishes labour considerably, requiring so little attention. There is no blackleading to do, no ironwork to polish, in most cases a wet cloth or a duster, and a few minutes of time only being necessary to clean it. That it greatly diminishes the quantity of smoke, and, owing to the construction of the fire-brick back, utilises the gases that, in an ordinary fireplace, pass up the chimney unconsumed. That it is as inexpensive as an efficient fireplace can be, and, finally, that it can be safely fixed on all floors of a building.

The London show rooms are at 28 Berners Street, Oxford Street, W.

## Figures and Tables

**Figure f1:**